# Three-Dimensional Proteome-Wide Scale Screening for the 5-Alpha Reductase Inhibitor Finasteride: Identification of a Novel Off-Target

**DOI:** 10.1021/acs.jmedchem.0c02039

**Published:** 2021-04-12

**Authors:** Silvia Giatti, Alessandro Di Domizio, Silvia Diviccaro, Eva Falvo, Donatella Caruso, Alessandro Contini, Roberto Cosimo Melcangi

**Affiliations:** † Department of Pharmacological and Biomolecular Sciences, Università degli Studi di Milano, via Balzaretti 9, 20133 Milano, Italy; ‡ SPILLOproject, via Stradivari 17, Paderno Dugnano, 20037 Milano, Italy; § Dipartimento Di Scienze Farmaceutiche, Università degli Studi di Milano, 20133 Milano, Italy

## Abstract

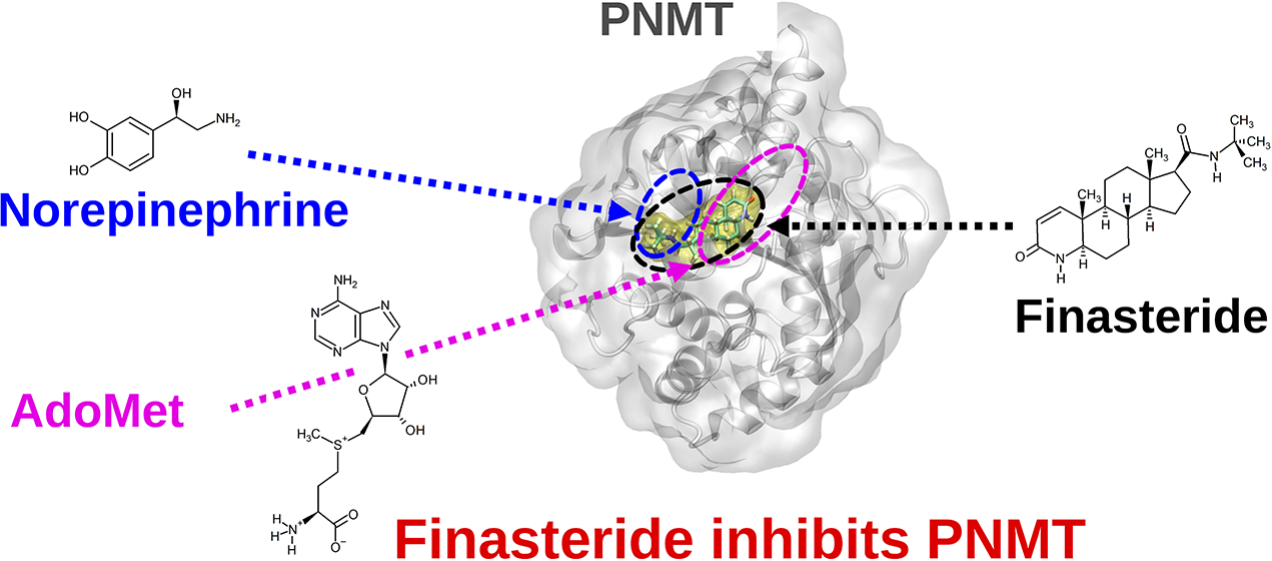

Finasteride, a 5-alpha reductase (5α-R) inhibitor, is a widely used drug for treating androgen-dependent conditions. However, its use is associated with sexual, psychological, and physical complaints, suggesting that other mechanisms, in addition to 5α-R inhibition, may be involved. Here, a multidisciplinary approach has been used to identify potential finasteride off-target proteins. SPILLO-PBSS software suggests an additional inhibitory activity of finasteride on phenylethanolamine *N*-methyltransferase (PNMT), the limiting enzyme in formation of the stress hormone epinephrine. The interaction of finasteride with PNMT was supported by docking and molecular dynamics analysis and by *in vitro* assay, confirming the inhibitory nature of the binding. Finally, this inhibition was also confirmed in an *in vivo* rat model. Literature data indicate that PNMT activity perturbation may be correlated with sexual and psychological side effects. Therefore, results here obtained suggest that the binding of finasteride to PNMT might have a role in producing the side effects exerted by finasteride treatment.

## Introduction

Finasteride (FIN), the prototypical 5-alpha reductase (5α-R) inhibitor, is a widely used drug with clinical relevance to treat androgen-dependent conditions, including benign prostate hyperplasia (BPH) and androgenetic alopecia (AGA). In 2017, US prescriptions for FIN were more than 9 million,^1^ with the global market projected to increase approximately 2.5% from 2020 to 2026. This compound will be increasingly requested due to the growing prevalence of BPH, linked to the rising of the geriatric population and to the surge in the demand due to increasing hair loss issues in males globally. Even though FIN is considered a safe medication, it causes negative side effects.^2,3^ However, the molecular mechanisms behind such adverse events are unknown. FIN blocks the conversion of testosterone into dihydrotestosterone (DHT) through competitive inhibition of type I and II 5α-R, with high selectivity for type II in humans. 5α-R type II (5α-R2) is present in the outer root sheaths of hair follicles, the epididymis, vas deferens, seminal vesicles, and prostate.^4−6^ Thus, in men, FIN reduces prostatic and serum DHT levels by more than 90 and 70%, respectively.^7^ Accordingly, most common complaints reported during FIN use are linked to sexual functions in both observational and clinical studies.^8−11^ In addition, depression, self-harm/suicide ideation,^12,13^ and sexual dysfunction^14^ have been observed during FIN treatment. In support, the American Food and Drug Administration Adverse Event Reporting System (FAERS) included the presence of such side effects.^15^ FIN can be consumed at a dose of 5 mg/day to treat BPH, while a dose of 1 mg/day is used for AGA. When the FAERS database is analyzed for FIN considering the dose, the retrieved side effects fall into three categories: sexual, psychological, and physical complaints.^16^ Interestingly, the lowest dose (1 mg/day) presented the highest number of reports. Among sexual function complaints, decreased or loss of libido, disorders of ejaculation, erectile dysfunction, testicular atrophy, orgasmic disorders, and hypogonadism were reported with the 1 mg/day dose. The same dose induced psychological alterations, such as an increase in self-harm, slow cognition and psychological pathology, changes in emotional effect, and sleep disturbances. In addition, when analyzing the physical domain, AGA subjects reported rash and metabolic abnormalities. However, patients with BPH that use a higher dose of FIN (*i.e.*, 5 mg/day) described gynecomastia as the most frequent adverse event due to pharmacological treatment.^16^ Overall, considering the side effects described, the blockade of the conversion of peripheral testosterone may not be the only factor responsible for the symptomatology reported after the 1 mg/day dose.

In the present study, a 3D proteome wide-scale *in silico* screening of a human protein database retrieved from the RCSB Protein Data Bank was performed using *SPILLO potential binding sites searcher* (SPILLO-PBSS, https://www.spilloproject.com),^17^ aimed at identifying alternative off-target interactions of FIN. In contrast to traditional structure-based approaches (e.g., molecular docking simulations), this software takes into account protein flexibility and recognizes potential targets and off-targets of any small molecule, even when the protein binding sites are strongly distorted (e.g., too open, occupied, or even fully closed) compared to a suitable conformation for the binding. SPILLO-PBSS has been recently used in similar tasks where it was able to identify off-target proteins (OTPs) of a new compound that was found to reduce migration and invasiveness in U87 glioblastoma cell lines.^18^ Then, the reliability of SPILLO-PBSS predictions was further assessed *in silico* by means of docking and molecular dynamics (MD) simulations aimed at testing the stability of FIN interactions with the predicted OTP, while providing insights on its binding mode. *In silico* experiments were followed by *in vitro* biochemical analysis that confirmed the interaction between FIN and the identified OTP. Finally, an innovative multilevel cross-organism transferability analysis (MCOTA) was performed to rationally choose a suitable model organism for subsequent molecular *in vivo* analysis that, in turn, further confirmed the proposed interaction.

Overall, our study identifies a novel FIN-interacting protein with the supposed ability to participate in the side effects observed in FIN users.

## Results

### Protein Database Screening and Ranking by SPILLO-PBSS

**Figure 1:**
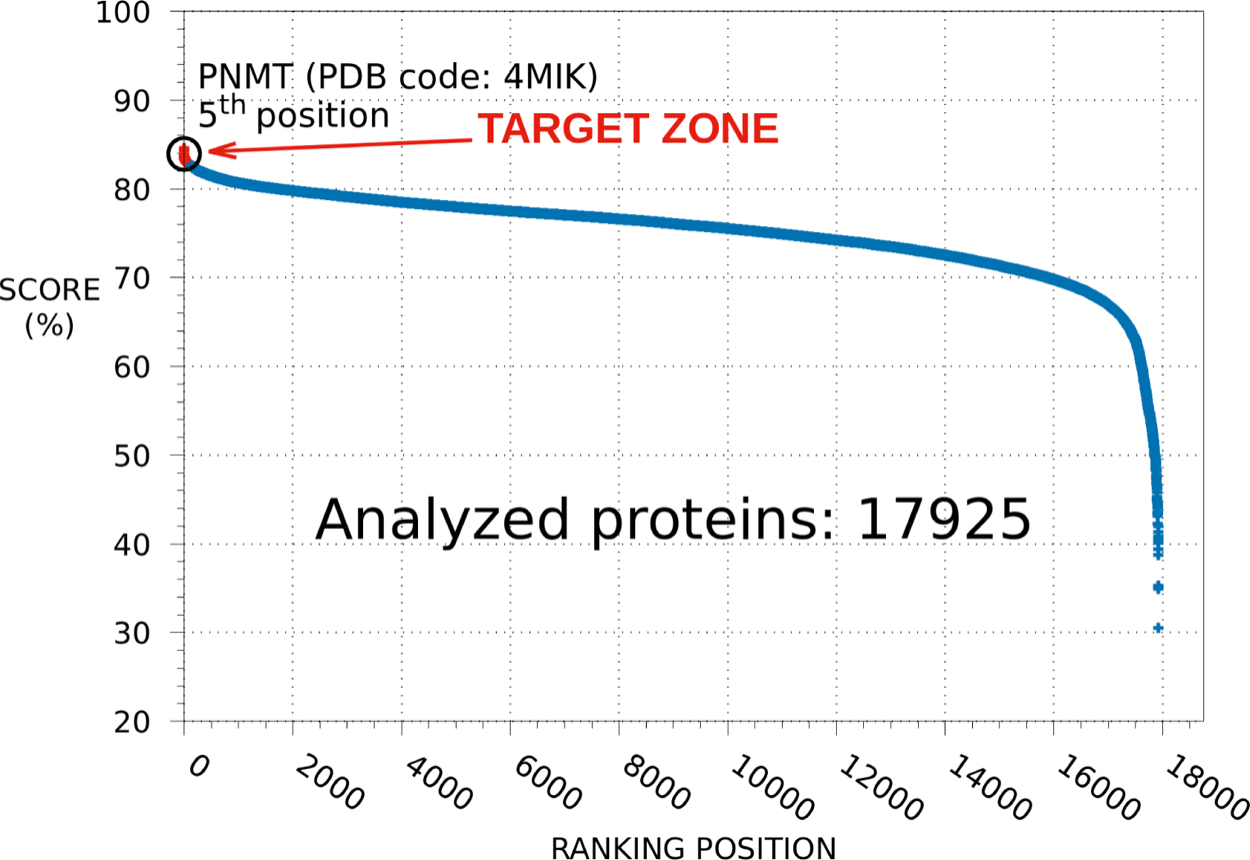
SPILLO-PBSS screening and ranking of the available structural proteome of *Homo sapiens*. Plot resulting from the SPILLO-PBSS screening and ranking of the available human structural proteome (17,925 holo- and apo-protein 3D structures from the RCSB Protein Data Bank in January 2019, without 100% sequence identity redundancies). Proteins are ranked in a decreasing order according to their score.

With the aim of identifying FIN OTPs that could correlate with the side effects reported in the FAERS database,^16^ the SPILLO-PBSS software was used to screen and rank the whole structural protein database of *Homo sapiens* available (January 2019). Results are summarized by the plot in Figure 1, in which points correspond to proteins ranked in a decreasing order according to their scores. The nonlinearity of the curve highlights the presence of a minority of proteins with scores clearly higher than all others, corresponding to the potential OTPs of FIN.

It should be noted that SPILLO-PBSS is designed to identify PBSs, where the interaction between the small molecule and the protein is mediated by proteogenic residues only, whereas other elements such as cofactors, nucleic acids, or cell membranes are currently not included in the model. Thus, it is not surprising that the enzyme 5β-R, an expected target for FIN,^19^ was not present in the top-ranked positions. Indeed, the binding site of 5β-R contains the NADP cofactor that plays an important role in mediating its interaction with FIN. The same considerations would also apply to 5α-R2, the therapeutic target of FIN, whose structure was not yet available in the PDB when the calculation was performed. However, the limitations of the model discussed above have no influence on the reliability of the OTPs found, in which the interaction with the drug is entirely mediated by amino acid residues.

Of the top-ranked potential OTPs identified by the software, we focused our attention on the phenylethanolamine *N*-methyltransferase (PNMT, PDB code: 4MIK). Indeed, a possible link was identified, as explained below, between a perturbation of the activity of this enzyme and some of the most common adverse effects of FIN.

### PNMT as a Potential OTP for FIN

Among the top-ranked potential OTPs of FIN, namely, in the fifth position out of 17,925 (SPILLO-PBSS score = 83.498%), PNMT (PDB code: 4MIK, UniProtKB AC: P11086) warranted further investigation. In fact, this off-target immediately caught our attention due to its close connection with the onset of the most common previously cited adverse effects of FIN, in particular, those related to sexual functions. For this reason, we decided to start our experimental validations from PNMT, while an in-depth analysis and validations for other top-ranked OTPs will be the matter of future studies.

**Figure 2:**
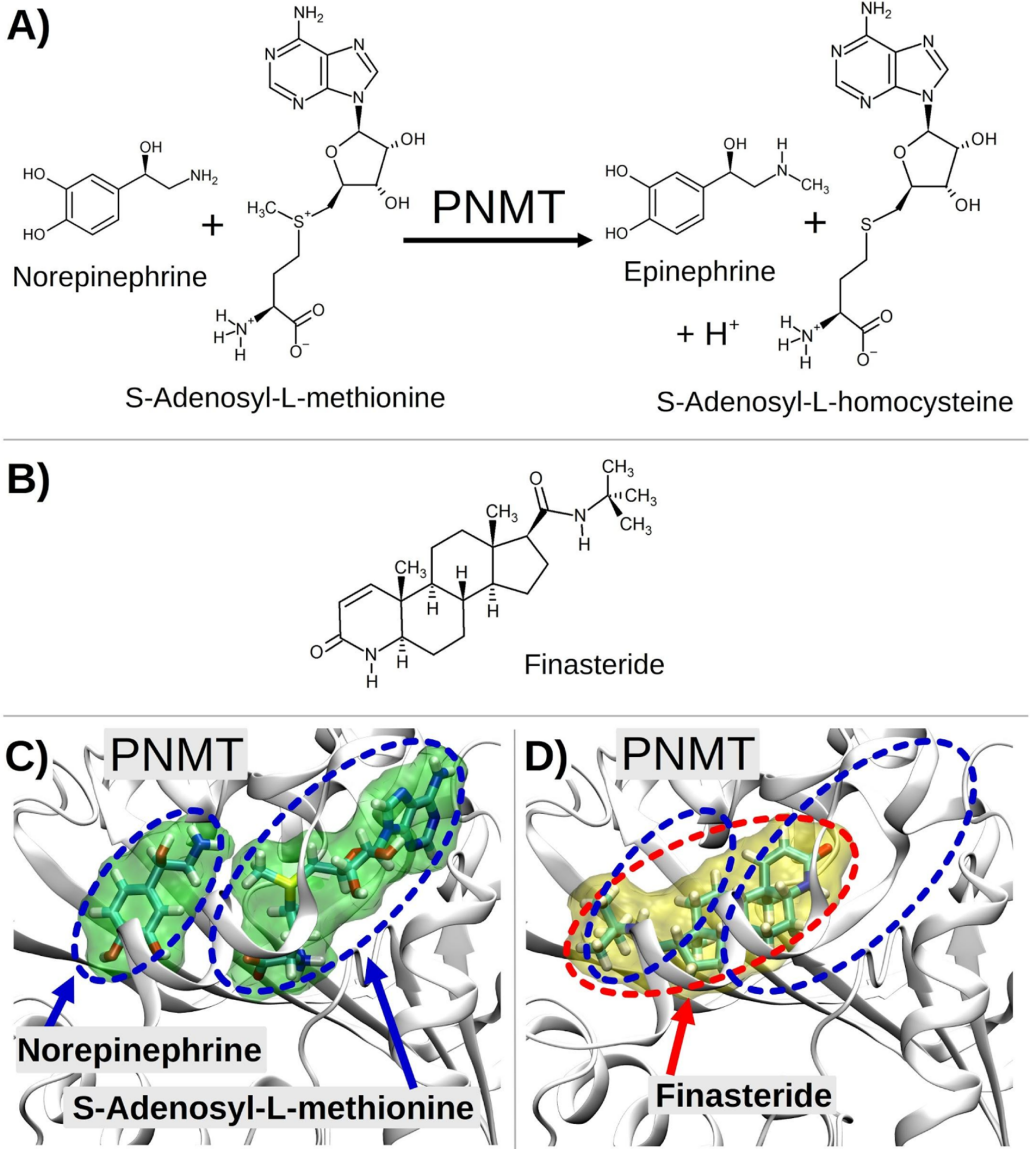
PNMT enzymatic reaction and competitive inhibition hypothesis. The enzymatic reaction (panel A) catalyzed by PNMT is reported along with the 2D formula of FIN (panel B). Norepinephrine and S-adenosyl-l-methionine (panel C—PDB ID: 3HCD) and FIN (panel D—PDB ID: 4MIK) within their corresponding binding sites in the same PNMT region are also shown, as obtained by X-ray diffraction and SPILLO-PBSS calculation, respectively (drawings rendered using VMD^31^).

PNMT principally catalyzes the conversion of norepinephrine to epinephrine (Figure 2A) and is mainly involved in the stress response.^20,21^ Epinephrine is strongly correlated with mood alteration and depression.^22−25^ In addition, several studies indicate a role for this hormone in the control of erection.^26−30^ Overall, these findings prompted us to consider PNMT as a potentially relevant factor in FIN side effects.

### Competitive Inhibition Hypothesis

A PBS for FIN similar to a reference binding site (RBS) (see Table S1 for a direct comparison between the FIN RBS and PBS) was identified within the PNMT 3D structure (PDB code: 4MIK). In particular, this PBS turned out to largely overlap with the catalytic site of the enzyme that, in this specific structure, is occupied by the bisubstrate inhibitor JIL. Within the PNMT binding site, norepinephrine and S-adenosyl-l-methionine are converted to epinephrine and S-adenosyl-l-homocysteine (Figure 2A), respectively. We thus hypothesized that FIN (Figure 2B) inhibits the catalytic activity of the enzyme by competing with norepinephrine and S-adenosyl-l-methionine for the same binding region (Figure 2C,D). PNMT structures shown in Figure 2C,D correspond to PDB files 3HCD and 4MIK, respectively. In the former, PNMT is cocrystallized with norepinephrine and S-adenosyl-l-homocysteine, which we virtually transformed into S-adenosyl-l-methionine for the sake of clarity.

### Docking and MD Simulations

It should be noted that SPILLO-PBSS is not a molecular docking program and is able to identify PBSs even when they are occupied by a ligand or in a closed conformation. Indeed, SPILLO-PBSS compares the 3D structure of the RBS with that of any PBS present in the target protein on a similarity basis. A tolerance (5.5 Å) is applied to take into account any conformational rearrangements that may occur to the binding site upon binding, thus implicitly considering some degree of side chain and backbone flexibility.^17,18^ As an output, SPILLO-PBSS provides a structure between the query ligand and the identified off-target protein, as is found in the PDB, so steric clashes might be present (see Figure S1), and the binding mode might not be ideal (see Table S2). For these reasons, the hypothetical complex between human PNMT (hPNMT) and FIN generated by SPILLO-PBSS was initially subjected to a geometry minimization. Then, we redocked FIN using MOE, and the top docking poses were refined by MD simulations. The top three poses of FIN (hereafter referred to as P1, P2, and P3) obtained by docking and the binding geometry obtained from the SPILLO-PBSS search (here referred as P0) were subjected to 200 ns of MD simulation in an explicit solvent, followed by binding energy calculations using the Nwat-MMGBSA method^32−34^ including 30 explicit waters around the ligand. The crystallographic complex described in 4MIK,^35^ where the bisubstrate ligand (referred as JIL in the PDB file) is bound to hPNMT, was also subjected to MD simulations using the same protocol adopted for FIN complexes. The binding energies reported in Table 1 show that the crystallographic ligand JIL is significantly more potent than FIN, which is expected considering that JIL fills the rather long binding pocket of hPNMT better than FIN (Figure S2A). However, all four binding poses obtained for FIN were stable enough during the 200 ns of MD simulation (Figure S3) to support the capability of FIN to bind hPNMT, as predicted by SPILLO-PBSS.

**Table 1:** Binding Energies Computed by Nwat-MMGBSA Analysis (Nwat = 30) of the Last 20 ns of the MD Trajectories of P0–P3 and JIL

	Δ*E*_bind_ (kcal/mol)
P0	–58.4 ± 3.7
P1	–55.3 ± 4.6
P2	–53.6 ± 3.9
P3	–55.8 ± 3.9
JIL	–75.3 ± 5.7

Additionally, pose P0 resulted in the most favored, even if the energy difference between the considered poses is not large enough to make an unequivocal choice among the predicted binding modes. However, by inspecting the representative geometry of the most populated cluster obtained from the analysis of P0–P3 MD trajectories (Figure S2), we observed that FIN is similarly oriented in P0–P2 with respect to the hPNMT binding pocket. Thus, poses P1 and P2 can be considered metastable states along the binding path eventually leading to P0, where FIN is deeply buried in the binding pocket. Conversely, a flipped orientation is observed in pose P3, with the δ-lactam ring occupying the same position of the *N*-*tert*-butylcarbamoyl moiety, as found for P0–P2, and vice versa. The most relevant hydrogen bonds (H-bonds) found by analysis of the MD trajectories of P0 and P1–P3 are reported in Tables S3 and S4, respectively. Considering P0, the most relevant H-bond, with an occupancy (occ %) higher than 94%, is observed between the amino group of the Lys57 side chain and the carbonyl group of the *N*-*tert*-butoxycarbonyl moiety of FIN (Table S3). A second interaction involving the same moiety is observed between the amido NH and the side-chain oxygen of Tyr35. Interestingly, a rather relevant H-bond bridged by a water molecule is found between the lactam carbonyl of the 4-aza-5α-androsten-3-one group and both the backbone carbonyl of Tyr27 and the side chain NH_2_ of Asn106 (occ % = 74.0). Hydrophobic interactions are also observed between the lipophilic core of FIN and Phe30, Trp123, Phe182, and Leu229 (Figure S2). P1 is similar to P0 in orientation with respect to the binding site but shifted outward (Figure S2). Indeed, the main H-bond is observed between the lactam NH and the side chain of Asp101 (occ % = 98.3, Table S4).

The same residue is also involved in a water-bridged interaction with the lactam C=O of FIN (occ % = 87.2), which also interacts with Phe102 NH through a water molecule (occ % = 40.2). Another relevant water bridge is observed between the *N*-*tert*-butoxycarbonyl and OH of Tyr85 (occ % = 76.8), while a water-mediated interaction with the side chain of Lys57 is only occasionally sampled (occ % = 11.8). P2 is the pose with the highest predicted binding energy and thus, is considered the least reliable. The lactam moiety of FIN overlays with the 6-aminopurine group of JIL that resides in the outermost zone of the binding site (Figure S2C). Only one direct H-bond is observed between the lactam C=O of FIN and the backbone NH of Val159 (occ % = 96.6, Table S4). Instead, three water-bridges are found. The first involves the lactam C=O and the side chain of Asp158 (occ % = 90.0), the others are found between the *N*-*tert*-butoxycarbonyl group and Pro32 and Phe182 (occ % = 80.9 and 72.5, respectively). Among the predicted poses, P3 is the one that differs the most since it is flipped along the longitudinal axes (Figure S2D) but still has a rather low binding energy (Table 1). However, this low binding energy can be explained by considering the chemical properties of FIN. Indeed, the two amide groups, separated by the hydrophobic polycyclic core, confer a rather symmetrical electrostatic distribution to FIN itself (Figure S4). Thus, an interaction profile with hPNMT that is comparable to that described for poses P0–P2 can also be observed for P3 (Table S4). H-bonds with Tyr27 and Tyr35 can indeed be observed but involving the carbonyl group of the *N*-*tert*-butoxycarbonyl moiety (occ % = 99.7) and the lactam NH (occ % = 87.5), respectively. Moreover, an additional H-bond is observed between the exocyclic carbonyl and the OH of Tyr40 (occ % = 42.8), while two water-mediated bridges are found between the lactam NH and Asp101 side chain (occ % = 35) and Phe102 backbone (occ % = 35).

**Figure 3:**
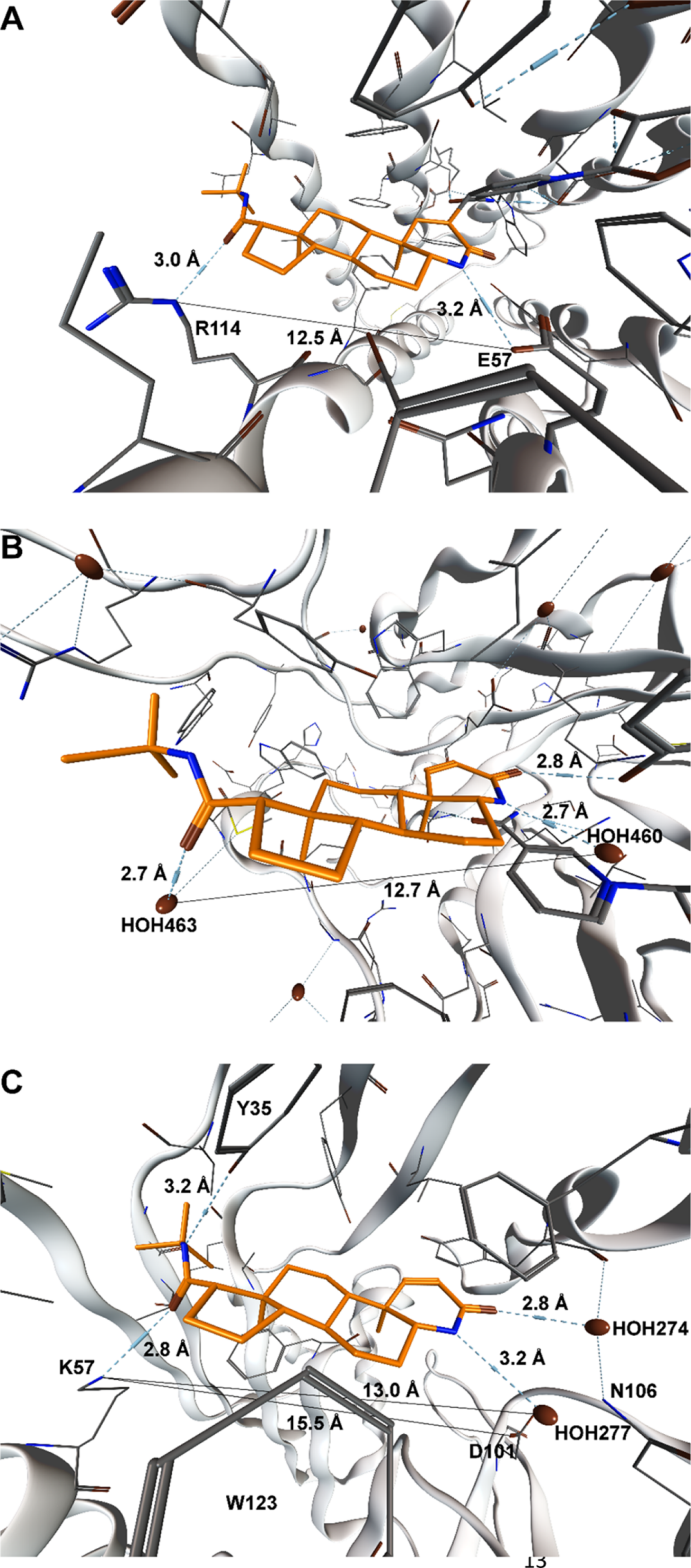
Complexes of FIN and 5α-R2, 5βR and hPNMT. FIN is depicted by a tube representation with carbon atoms colored in orange. Panel A represents the complex of the drug with 5α-R2 (PDB ID: 7BW1); Panel B represents the complex of the drug with 5βR (PDB ID: 3G1R); panel C represents the complex of the most favored pose of the drug bound to hPNMT, as obtained by simulations. H-bonds involving FIN and receptor or solvent atoms are represented by dashed light-blue lines including a cylinder, whose length is proportional to the H-bond estimated strength. Donor–acceptor distances are reported for H-bonds between FIN and receptors. Selected geometrical distances between anchor points (enzyme atoms or bridging waters) of FIN to each system are represented by plain black lines along with corresponding values.

To better understand how FIN can bind to hPNMT, a comparison was made between our predictions and the experimental binding modes determined for FIN bound to 5α-R2, whose crystal structure was recently released,^36^ and 5β-R (Figure 3). In all three structures, the binding of FIN with the enzyme is mediated by several hydrophobic contacts and by H-bonds involving the cyclic and exocyclic amido groups. In the 5α-R2 complex (Figure 3A), direct H-bonds are found between Arg114 (donor) and the FIN *N*-*tert*-butylcarbamoyl carbonyl (acceptor) and between Glu57 (acceptor) and the FIN δ-lactam NH (donor). A distance of 12.5 Å is measured between Arg114 and Glu57.

These direct H-bonds between FIN and the receptor are not observed with 5β-R (Figure 3B). Although H-bonds are not essential for binding, when hydrophobic interactions are possible, they are important for ligand efficiency and specificity.^37,38^ However, crystallographic waters are found making H-bonds with the same donor and acceptor groups of FIN, and the distance measured between the two water oxygens is 12.7 Å. When studying the hPNMT structure, a cationic and an anionic residue, Lys57 and Asp101, respectively, were found able to interact with FIN similarly to 5α-R2. In particular, Lys57 directly interacts with the *N*-*tert*-butylcarbamoyl carbonyl of FIN, whereas Asp101 interaction with the drug is mediated by a bridging water connecting the FIN NH group to the Asp101 side chain. Here, the distance between Lys57 and Asp101 is 15.5 Å (Figure 3C), 3.0 Å larger than the distance measured between 5α-R2 Arg114 and Glu57, but the interaction is not lost. Indeed, the distance measured between the Lys57 and water HOH277, directly connected to FIN NH, is of 13.0 Å. Within the second most favored pose (P1) predicted for FIN bound to hPNMT (Figure S2B), Lys57 and Asp101 are at a distance (15.8 Å) similar to that measured in P0, but in P1, FIN is directly bound to Asp101 and a water bridge mediates its interaction with Lys57.

### *In Vitro* hPNMT Activity Assay

**Figure 4:**
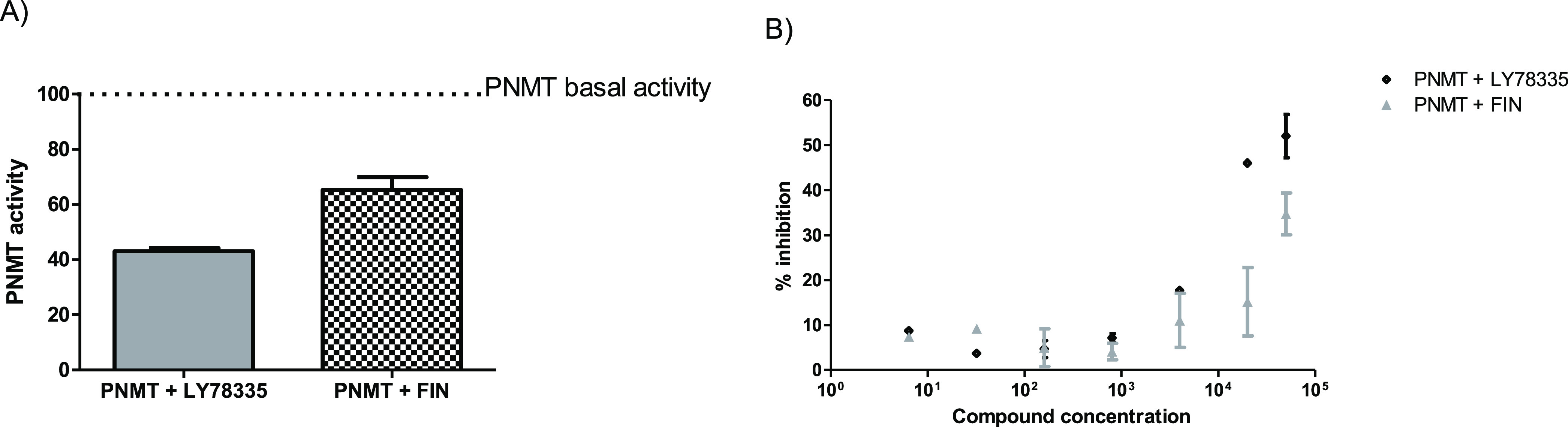
hPNMT activity. Human recombinant enzyme activity was detected by fluorescence in the presence of a vehicle (DMSO—basal PNMT activity) of LY78335 or FIN. Panel A: PNMT activity in the presence of a high-affinity inhibitor LY78335 (50 μM) or FIN (50 μM). The columns represent the mean ± SEM of duplicate reactions. Panel B: dose–response curve in the presence of a high-affinity inhibitor LY78335 (black rhombus; from 6.4 nM to 50 μM) or FIN (gray triangle; from 6.4 nM to 50 μM). The symbols represent the mean ± SEM of duplicate reactions.

To verify whether FIN might make contact with hPNMT enzyme through an inhibitory interaction, an *in vitro* inhibition assay was set up. First, the basal hPNMT activity (between 5.43 and 9.28 pmol/min/μg) was obtained and was in line with the expected activity indicated by the supplier. Then, FIN was tested at the concentration of 50 μM to evaluate the inhibitory activity *in vitro* (Figure 4). In parallel, the known PNMT inhibitor LY78335^39^ was tested at the same concentration (50 μM) as a positive control. The concentrations tested were selected based on previous protocols evaluating the unknown compounds’ inhibitory activity on PNMT activity.^39,40^ In these reports, inhibitors were tested at the same concentration of the methyl donor (S-adenosyl-l-methionine). Thus, based on the protocol indicated by the supplier, the selected compound’s concentration was 50 μM, as S-adenosyl-l-methionine.

As reported in Figure 4A, LY78335 inhibited hPNMT activity by nearly 60%. The same concentration of FIN (*i.e.*, 50 μM) was able to reduce hPNMT activity by more than 30%.

Based on the published Ki,^39^ in the present assay, LY78335 was able to inhibit less than expected PNMT activity. On the other hand, it is important to recall that the published assays^39,40^ were conducted under very different experimental conditions, and this affected the final inhibitory activity. Moreover, this situation is very far from the physiological condition in adrenal glands. Thus, the concentrations here reported are intended only for a comparison of the inhibitory ability of the compounds in this particular assay and do not want to describe any physiological situation. To test if the assay we designed is reliable, a dose–response curve was prepared (Figure 4B). As reported, several concentrations (from 6.4 nM to 50 μM) of LY78335 and FIN were able to inhibit PNMT activity to different extents. The results indicated that LY78335 inhibits with higher potency PNMT activity than FIN, as expected.

### Rational Design of Animal Testing

To evaluate if the described *in vitro* inhibitory effect was also maintained *in vivo*, further animal tests have been rationally designed in compliance with the guidelines provided by the 3R principles.^41^ In fact, the precise knowledge of the human SPILLO-PBSS-predicted OTP for FIN, along with the structural details concerning its PBS provided by the software, made it possible to perform an innovative MCOTA aimed at identifying the most suitable model organism for the *in vivo* tests from a pool of possible model organisms (*i.e.*, *Caenorhabditis elegans*, *Danio rerio*, *Drosophila melanogaster*, *Mus musculus*, and *Rattus norvegicus*). In particular, a basic check for the presence of the PNMT gene allowed to primarily discard *C. elegans*, *D. rerio*, and *D. melanogaster* as the PNMT gene is excluded from their genome. Then, an overall comparison between the hPNMT sequence and PNMT sequences of *M. musculus* and *R. norvegicus*, followed by a local 3D structural comparison between their PBSs for FIN, provided a positive assessment for the use of these two model organisms in our tests, with a slight advantage for *R. norvegicus*. A final SPILLO-PBSS calculation was then performed to confirm that *R. norvegicus* and *M. musculus* PNMTs belong to the target zone of the global FIN OTPs ranking. Thus, only these two structures were included in the calculations performed previously, within the human protein database. Namely, *R. norvegicus* and *M. musculus* PNMTs ranked fifth (causing the hPNMT to scale down to sixth) and seventh (out of 17,927), respectively. Thus, analysis by MCOTA determined that *C. elegans*, *D. rerio,* and *D. melanogaster* were not suitable for our tests, while indicating that *R. norvegicus* and *M. musculus* were rather reliable and nearly equivalent (see Table S5). *R. norvegicus* was chosen because of the team’s experience with this model organism during previous studies concerning FIN.^42,43^

### Catecholamine and PNMT Adrenal Content

**Figure 5:**
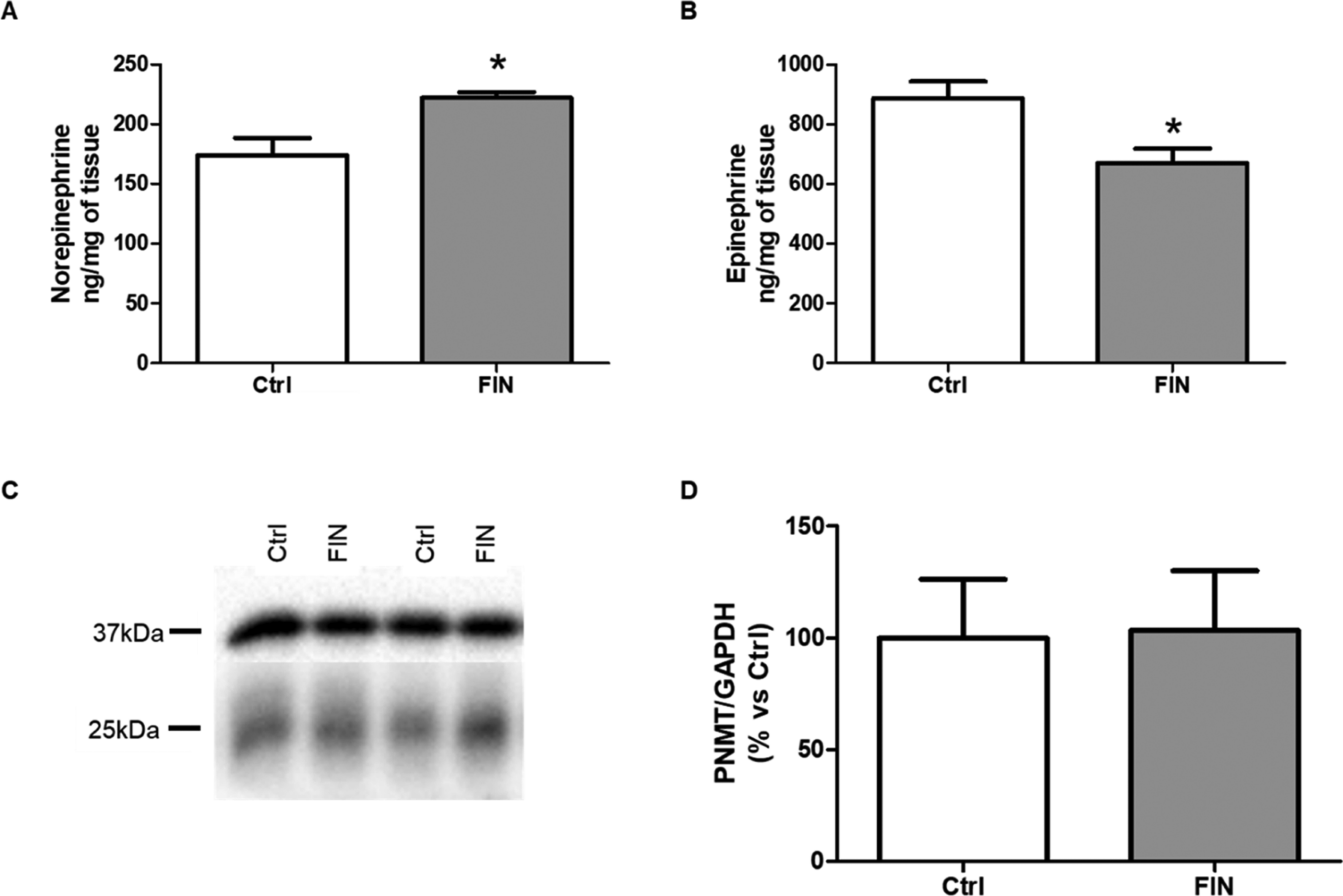
Adrenal catecholamine and PNMT protein levels. Catecholamine levels were detected by liquid chromatography tandem mass spectrometry analyses in control (Ctrl; *n* = 6) and FIN-treated animals (FIN, *n* = 6). Adrenal PNMT protein levels were detected by western blotting in control (Ctrl; *n* = 6) and FIN-treated animals (FIN, *n* = 6). Panel (A) norepinephrine levels; panel (B) epinephrine levels; panel (C) representative blot of PNMT (25 kDa) and GAPDH (37 kDa); panel (D) quantification of PNMT protein levels. The columns represent the mean ± SEM after normalization for the starting tissue (panel A and B) or for GAPDH (panel D). Data were analyzed by Student’s *t*-test. *p ≤ 0.05 vs Ctrl.

Based on the results obtained by MCOTA, adult male Sprague-Dawley rats were treated with FIN, as reported.^42,43^ As described previously,^43^ the dose and timing applied in this experiment have been selected based on the ability to affect DHT plasma levels and prostate gland weights. Indeed, plasma DHT levels and prostate gland weights were both reduced in FIN-treated rats, thus indicating an effective FIN treatment (^43^ and data not shown). As reported above, PNMT enzyme is responsible for epinephrine production and the adrenal gland represents the main source of this hormone.^44^ Therefore, epinephrine and norepinephrine levels were evaluated by liquid chromatography tandem mass spectrometry analyses in the adrenal glands of FIN-treated rats and compared to vehicle-treated animals. As reported in Figure 5, norepinephrine levels significantly increased after drug treatment (Figure 5A), while those of epinephrine decreased in a statistically significant way (Figure 5B). The data obtained are in line with the hypothesis of a reduced enzymatic activity due to FIN inhibition.

To determine if the FIN effect on PNMT was due to a deficit in its protein expression levels, western blotting was performed. No differences in protein levels of PNMT enzyme were detected in the adrenal glands of FIN-treated rats in comparison to vehicle-treated animals (Figure 5C,D). Overall, the data obtained after *in vivo* FIN treatment indicate a decreased conversion of norepinephrine into epinephrine in the adrenal glands of drug-treated rats, reasonably due to a reduced enzymatic activity.

## Discussion and Conclusions

To our knowledge, this is the first report demonstrating that other proteins besides 5α-R may be OTPs of FIN. The 3D proteome-wide scale SPILLO-PBSS *in silico* screening identified hPNMT as a top-ranked protein possibly able to interact with the drug. Furthermore, SPILLO-PBSS showed that the binding site of FIN within hPNMT overlaps with that of norepinephrine and S-adenosyl-l-methionine. Therefore, it is possible that FIN, competing with physiological substrates, may reduce the catalytic activity of PNMT in converting norepinephrine to epinephrine. Docking and MD simulations elucidated possible binding modes of FIN within the hPNMT binding site and provided further elements supporting the stability of the predicted interaction. The *in silico* predictions were confirmed by a biochemical assay, indicating an inhibition of hPNMT by FIN *in vitro*. To evaluate whether this effect is maintained *in vivo*, animal tests were designed by MCOTA in compliance with the 3R principles. Thus, the ability of FIN in inhibiting PNMT was tested *in vivo* by treating male rats and analyzing norepinephrine and epinephrine levels in the adrenal gland by liquid chromatography–tandem mass spectrometry (LC–MS/MS). In support of our hypothesis, results revealed a reduction in the levels of epinephrine, with a concomitant increase of norepinephrine levels, coupled to no effect on PNMT protein levels. These findings suggest that the altered hormonal levels were due to a reduction of the enzymatic activity rather than to decreased levels of PNMT.

When this study was conducted, the crystal structure of FIN complexed with its principal target 5α-R2 was not available in the PDB, where only the complex with 5β-R was present.^19^ The former enzyme is irreversibly inhibited by FIN that forms a covalent bond with the NADPH cofactor. A subnanomolar affinity was determined for the NADP–dehydrofinasteride (NADP–DHF) adduct, thus explaining the irreversible inhibition. Conversely, FIN inhibits 5β-R with a low μM affinity,^19^ and no covalent adduct is formed. A crystal structure of 5α-R2 complexed to NADP-DHF has been recently released,^36^ allowing a comparison between the experimental binding modes determined for FIN bound to either 5α-R2 or 5β-R and the predicted binding mode for hPNMT (Figure 3). In the three structures, it can be observed that FIN binding with the enzyme is mediated by several hydrophobic contacts and by H-bonds involving the cyclic and exocyclic amido groups. Thus, it is possible that a distance of 12.5 Å between a H-bond donor and a H-bond acceptor is needed for optimal ligand–receptor interactions. This distance is met for 5α-R2 only, the enzyme for which FIN is highly selective. The same is not observed in 5β-R, but FIN can still bind, even if with micromolar affinity, due to hydrophobic interactions and water-mediated H-bonds. Concerning hPNMT, the H-bond donor and acceptor are at the right position, but their distance is too long for optimal interactions. However, water can accommodate this distance by forming bridges between FIN and Lys57 or Asp101.

PNMT represents the final step in the pathway generating epinephrine, thus identifying adrenergic cells. The PNMT expression has been found in different regions of the nervous system, including the medulla oblongata, brainstem, locus coeruleus, diencephalon, and sympathetic ganglia,^45^ as well as in other tissues,^70−75^ such as in the heart, spleen, thymus, retina,^46^ and mainly, in chromaffin cells of the adrenal medulla.^44^

While epinephrine is responsible in controlling the peripheral stress response, norepinephrine seems to have major relevance in the brain.

Focusing on the peripheral actions of these two catecholamines, it is important to recall the involvement of epinephrine in the “fight or flight” response.^47^ To response a stressful situation (emotional, environmental, or physical), the adrenal medulla rapidly releases the stored epinephrine into the general circulation.^21^ This, in turn, leads to the subsequent release of cortisol in humans. The cooperation among these hormones produces different physiological responses involving central and sympathetic nervous system functions. Indeed, norepinephrine and epinephrine are involved in the increase in the heart rate and blood pressure. Metabolically, they increase fat metabolism and glucose blood levels to sustain the energy demand of the body. Exogenous epinephrine administration increases blood glucose by inhibition of insulin release, stimulation of glucagon release, hepatic glycogenolysis, and hepatic and renal gluconeogenesis.^48^ Both norepinephrine and epinephrine stimulate β3 receptors to promote fat metabolism, while epinephrine stimulates the thermogenic response in humans.^49^

The noradrenergic system is also involved in the regulation of male sexual functions.^50,51^ The central control of erection is linked to ascending signals to the brain and descending ones to the spinal cord.^51^ In addition, adrenergic innervation is strongly present in penile arteries and veins and in cavernosal smooth muscle.^52^ In support, vascular control by the autonomic nervous system regulates erection.^53^ The first phase of the erection cycle in humans is the flaccid state; then, upon stimuli, the penis reaches tumescence until complete rigidity, to conclude with the detumescence phase, back to the initial flaccid condition. Adrenergic control is involved in all erection phases.^54^ Indeed, norepinephrine release in the penis induces the flaccid state by contracting the trabecular smooth muscle.^55^ Accordingly, in healthy men, a reduction in norepinephrine levels is associated with penile tumescence and erection, while an increased level of this hormone is associated with the transition from rigidity to detumescence.^26^ Thus, norepinephrine is involved in transforming the penis from the erect to flaccid state.^56^ In contrast to norepinephrine, epinephrine levels are increased in the tumescence phase in relation to the flaccid condition and then decreased in the rigid and detumescence phases.^27^ Thus, these two hormones have opposite functions in penile erection. In human psychogenic and neurogenic erectile dysfunctions, the rigidity phase cannot be achieved. Interestingly, in these conditions, plasma norepinephrine levels are high in flaccidity, tumescence and detumescence phases, suggesting an impairment in adrenergic signals.^27^ In summary, the balance of epinephrine and norepinephrine is crucial for achieving an erection.^76,77^ Thus, based on the results we obtained, it is possible that FIN administration, by affecting PNMT enzymatic activity, is involved in the sexual problems reported by FIN users.

Interestingly, norepinephrine may also influence the gut microbiota, the community of bacteria that reside in the mammalian gut and have profound effects on physiology and disease.^57^ In particular, gut microbiota is involved in essential functions, such as digestion, vitamin production, and defense against pathological bacteria.^25^ On the other hand, alterations of microbial composition in the gastrointestinal tract are associated with many pathological situations, including depression,^58^ anxiety,^59^ and other neurological pathologies.^60,61^ A direct interaction among the host and gastrointestinal bacteria has been proposed, with catecholamines as major modulators of this communication.^62^ Indeed, given that the communication is bidirectional, many studies have focused on gut bacteria production of catecholamines and their influence on brain functions.^25,62^ However, how host-produced catecholamines can influence the gut microbiome has been less explored. *In vitro* evidence suggests that norepinephrine promotes the growth of Gram-negative bacteria and, in general, increases virulence and facilitates bacterial invasion.^63^ In addition, Houlden and colleagues described altered microbiota composition in an experimental model of stroke, where increased norepinephrine levels have been observed.^64^ Interestingly, FIN administration altered gut microbiota composition in male rats.^42^ Whether the impaired microbial composition was a consequence of presumed altered levels of norepinephrine or rather a direct action of FIN on the gut community remains to be assessed. However, the possible influence of catecholamine in the rat experimental model could not be ruled out.^78−83^ In this context, in relation to the well-known gut microbiota–brain axis, the involvement of altered microbiota and depression–anxious conditions reported by FIN users should be taken into account.

In conclusion, data presented here indicate that the 5α-R inhibitor FIN is also able to interact with PNMT. This concept is supported by 3D proteome wide-scale screening, by docking and MD simulations, by an *in vitro* biochemical assay, and *in vivo* analysis. We believe that the present findings may help in explaining the various side effects reported by FIN users, in particular those related to sexual function and gut-microbiota alterations. In future studies, it will be critical to further explore the consequences of FIN–PNMT interaction. For instance, recent observations identified PNMT genetic variants and polymorphisms in human subjects. Interestingly, some haplotypes resulted in decreased activity or accelerated degradation and different abilities to produce epinephrine in the basal condition or during exercise.^65,66,84,85^ Probably, carriers of PNMT variants might react differently to FIN administration, inducing different responses. Finally, considering that FIN may cross the blood–brain barrier, it will be crucial to explore the possible influence of this drug on brain PNMT^67−69^ and the subsequent pathology.

## Experimental Section

### Drugs and Reagents

FIN (Merck Life Science S.r.l., Milano, Italy, Catalog #F1293), dopamine-1,1,2,2-*d*_4_ hydrochloride (Merck Life Science S.r.l., Milano, Italy, Catalog # 655651), MES buffer (Merck Life Science S.r.l., Milano, Italy), dl-Norepinephrine hydrochloride (Merck Life Science S.r.l., Milano, Italy, Catalog # A7256), S-adenosyl-l-methionine (AdoMet) (Merck Life Science S.r.l., Milano, Italy, Catalog # A7007), reduced glutathione, and ThioGlo 3 fluorescent thiol reagent (Covalent Associates, Inc., Catalog # T003) were obtained. All recombinant human enzymes and the PNMT inhibitor LY78335 (Catalog # 4060) were purchased from Bio-Techne, Milano, Italy: PNMT (rhPNMT, Catalog # 7854MT), adenosyl homocysteinase/AHCY (rhAHCY, Catalog # 6466AH), adenosine deaminase/ADA (rhADA, Catalog # 7048AD).

The purity of LY78335 and FIN compounds was declared to be 99 and ≥98%, respectively, by the manufacturers.

Antibodies: PNMT (Novus Biologicals, Centennial, CO, USA, NBP2-00688); GAPDH (Santa Cruz, Dallas, Texas, US, sc-25778).

### Protein Database Preparation

The protein database used for SPILLO-PBSS screening was generated by collecting all human protein 3D structures available in the RCSB Protein Data Bank (update January 2019) experimentally solved by either X-ray diffraction or solution NMR, excluding 100% sequence identity redundancies. It included 17,925 holo- and apoprotein 3D structures. Biological assemblies for proteins showing multimeric structures were then generated by the MakeMultimer program (http://watcut.uwaterloo.ca/tools/makemultimer/index) according to the BIOMT transformation matrices included in the PDB files. For multimodel PDB files from solution NMR experiments, only the first model was included in the database. No further refinements of the protein structure were conducted to improve the quality of protein 3D structures in the database.

### RBS Generation

The RBS used by SPILLO-PBSS to search the protein database for potential OTPs of FIN was obtained using molecular modeling techniques and the standard RBS generation protocol described in the SPILLO-PBSS paper.^17^ It included 18 amino acid residues directly interacting with the drug without any cofactor or water-mediated contact. The detailed amino acid composition of the RBS is reported in Table S1.

### *In Silico* Screening and Ranking of the Protein Database

An unbiased and systematic search for FIN PBSs within all protein 3D structures included in the database was performed by SPILLO-PBSS. Calculations were performed using a rotation step of 30° and a grid spacing of 2.0 Å, with the geometric tolerance set to 5.5 Å. SPILLO-PBSS analyzed all proteins of the database, and a ranking of the PBSs for the drug was obtained for each protein and stored by the program. Then, the protein database was ranked according to the highest PBS score, representing the highest similarity to the corresponding RBS, obtained from each analyzed protein 3D structure.

### Docking

The receptor model used for docking was derived from the PDB entry 4MIK(35) that describes the crystal structure of hPNMT in complex with an inhibitor (hereafter referred to as JIL, as named in the PDB file) at a resolution of 1.95 Å. Chain B, where the N-terminus is better resolved, was used to prepare the model. Crystallographic water molecules and ligands other than the inhibitor were initially removed. Then, the structure was processed by the *QuickPrep* tool of MOE,^86^ using the default settings. *QuickPrep* automatically corrects any inconsistency of the PDB file and protonates the complex at pH = 7. The last step consists of a tethered geometry minimization, where tethers (10 kcal/mol/Å) are applied to the receptor atoms up to 8 Å from the ligand, while keeping the farthest ones fixed. The receptor coordinates were then saved for further studies. Docking was initially tested on the JIL ligand using the MOE software. The optimized protocol used the Triangle Matcher algorithm^86^ with a timeout of 30,000 s and many returned poses increased to 100,000. A first scoring was performed using the London dG function, and rescoring was done on the top 100 poses using the induced fit method coupled to the GBVI/WSA dG function.^87^ The top ranked pose showed a root-mean-squared deviation (RMSD) to the crystallographic ligand of 1.9 Å and was then considered reliable enough to be applied to the docking of FIN.

### Molecular Dynamics

After visual inspection, the top three docking poses of hPNMT complexed to FIN (hereafter referred to as P1, P2, and P3, respectively, according to the ranking obtained from docking), with the structure of the complex directly obtained from the SPILLO-PBSS run (hereafter referred to as P0), were subjected to MD simulations. The model of JIL bound to hPNMT, obtained as described above, was also subjected to the same protocol as a reference. All MD simulations and analyses were performed using the Amber18 and AmberTools18 software packages.^88^ Charge parameterization of JIL and FIN was performed by the antechamber using the restrained electrostatic potential (RESP) method.^89^ After the solvation of the complexes by an octahedral TIP3P box^90^ up to 10 Å from the solute, the charge of each system was neutralized by adding four Na^+^ ions. MD simulations were then conducted using *pmemd.cuda*.^91^ The *ff14SB*(92) and *gaff*(93) force fields were adopted for protein and ligands, respectively. A protocol consisting in multiple equilibration steps up to a final temperature of 300 K at a constant volume and temperature (*NVT*) and constant pressure and temperature (*NPT*) was adopted as detailed in previous works,^94,95^ followed by 200 ns of *NPT* production run. After analysis of the variation of the RMSD versus time during the whole trajectory (Figure S1), H-bond (donor–acceptor distance cutoff = 3.5 Å; donor-H-acceptor angle cutoff = 135 deg) and clustering (five clusters based on mass-weighted RMSD were generated using the average linkage algorithm) analyses were performed using *cpptraj*(88) on the last 20 ns of each MD trajectory, where the RMSD was better converged. MD simulations were performed twice, and comparable results were obtained. Binding energies were computed using the Nwat-MMGBSA method.^32−34^ As suggested by the authors, 30 explicit water molecules, that are the closest to ligand atoms in each selected frame, were included in the calculation as part of the receptor (Nwat = 30), while the entropic contribution to the binding energy was neglected.

### *In Vitro* hPNMT Activity Assay

A first set of experiments was performed to evaluate the *in vitro* enzymatic hPNMT activity. The assay was performed by following the protocol developed by R&D, Bio-Techne, available on-line, to measure PNMT S-adenosyl-l-methionine-dependent ability to transfer a methyl group to norepinephrine. The assay is based on the measurement of the thiols present after PNMT methylation of norepinephrine; thus, it represents an indirect quantification of PNMT activity. Then, an *in vitro* hPNMT inhibition assay was set, and the protocol was modified as follows. In brief, the known PNMT inhibitor LY78335, FIN or vehicle dimethyl sulfoxide (DMSO) were preincubated separately for 30 min at 25 °C, with the hPNMT enzyme. For each experiment, a duplicate reaction of experimental points and a blank (without hPNMT), hereafter referred to as “reactions”, was performed. Then, a substrate mixture containing 50 μM (final concentration) AdoMet and 125 μM dl-norepinephrine hydrochloride was incubated to all experimental points for 30 min at 25 °C. The reactants were boiled at 95–100 °C for 5 min and then cooled in ice for 3 min. An equal volume of the hydrolysis mixture (6.25 μg/mL rhAHCY and 0.625 μg/mL rhADA) was added to reactions. After 1 h incubation at 37 °C, the samples were plated into a black multiwell in duplicate and 50 μM of ThioGlo 3 fluorescent thiol reagent was added to reactions and to the standard curve points prepared with glutathione. The sealed plate was incubated at RT for 5 min in the dark, and excitation and emission wavelengths of 380 and 445 nm (top read), respectively, were read with an EnSpire workstation (PerkinElmer). For the dose–response curve, the following (final) concentrations have been tested: 6.4 nM, 32 nM, 160 nM, 800 nM, 4 μM, 20 μM, and 50 μM.

The specific activity (pmol/min/μg) was calculated as follows: thiol produced (pmol)/[incubation time (min) *x* amount of enzyme (μg)]. All absorbance values were adjusted for the blank measurement, and then, the values obtained were interpolated with the standard curve. The result obtained for each sample represents the thiol produced. Since each experiment was performed as a duplicate, the final result was the mean of the two values obtained. Finally, the specific activity of PNMT + vehicle was put at 100%, and the activity of PNMT with LY78335 or FIN was calculated in relation to PNMT + vehicle.

### Multilevel Cross-Organism Transferability Analysis

The MCOTA consisted of the following checks and analyses performed on a pool of possible model organisms (*i.e.*, *C. elegans*, *D. rerio*, *D. melanogaster*, *Mus musculus*, and *R. norvegicus*):

(i) Basic check (Table S5, column I). A basic check for the presence/absence of the PNMT gene in the considered model organisms was performed by the UniProtKB webserver.
(ii) Overall protein sequence comparison (Table S5, column II). An assessment of the degree of similarity and identity (calculated by the EMBOSS Needle webserver https://www.ebi.ac.uk/Tools/psa/emboss_needle/) between the hPNMT (UniProtKB: P11086) and the same protein in *M. musculus* (UniProtKB: P40935) and *R. norvegicus* (UniProtKB: P10937) provided a preliminary evaluation of these two model organisms. However, although this analysis is informative regarding an overall comparison between sequences, it cannot detect local differences in the PBS amino acid composition, which may affect the way the PNMT from different organisms interact with FIN.
(iii) Local 3D structural comparison (Table S5, column III). A search (calculations carried out by the SPILLO-PBSS software) for the PBSs on the PNMT of *M. musculus* and *R. norvegicus* allowed to assess their similarity with the PBS found on the hPNMT and to point out any local differences (e.g., a different amino acid composition of the PBSs) that may affect their interaction with FIN. After a first visual check aimed at confirming the same position of the PBSs on the various protein structures and the spatial orientation of FIN within the PBSs, the SPILLO-PBSS scores were compared, which simultaneously consider several features of the PBSs, including their amino acid composition and the stabilizing contribution of each amino acid residue to the ligand binding.^17^ Since no PNMT 3D structure of the two model organisms was available in the RCSB Protein Data Bank, it was first necessary to generate the homology models, calculated using the SWISS-MODEL webserver^96^ using the hPNMT 3D structure (PDB code: 4MIK) as a template; then, the SPILLO-PBSS software was applied (with the same settings previously used for the protein database screening) to analyze the PNMT 3D structures of *M. musculus* and *R. norvegicus*.
(iv) Proteome-scale ranking-position evaluation (Table S5, column IV). The ranking positions of PNMT of *M. musculus* and *R. norvegicus* have been evaluated with respect to the interaction with FIN. This made it possible to assess whether they belong to the target zone and how they eventually rank with respect to hPNMT. The new ranking including the two model organism PNMTs was obtained by integrating the SPILLO-PBSS scores calculated in the previous step into the original ranking of the protein database.

### Animals and Treatments

Male Sprague-Dawley rats (150–175 g at arrival, Charles River Laboratories, Lecco, Italy) were housed in the animal care facility of the Dipartimento di Scienze Farmacologiche e Biomolecolari (DiSFeB) at the Università degli Studi di Milano, Italy. All animals were kept in standard rat cages with food and tap water available ad libitum and under controlled temperature (21 ± 4 °C), humidity (40–60%), room ventilation (12.5 air changes per hour), and light cycles (12 h light/dark cycle; at 7 a.m./off 7 p.m.). The rats were acclimated to the new environment for 7 days, and then, after they were 2 months old, they were randomly divided into two experimental groups: (i) control, vehicle-treated rats (Ctrl) and (ii) FIN-treated rats. All experimental procedures were performed in strict accordance with the Italian and EU regulation on animal welfare and were previously approved by our institutional animal use and care committee (OPBA office) by the Italian Ministry of Health (authorization 1083/2015-PR) and followed national (D.L. no. 26, March 4, 2014, G.U. no. 61 March 14, 2014) and international laws and policies (EEC Council Directive 2010/63, September 22, 2010: Guide for the Care and Use of Laboratory Animals, United States National Research Council, 2011).

FIN and vehicle treatments have already been described.^43^ In brief, FIN (1 mg/rat/day) dissolved in a vehicle (5% ethanol in sesame oil) or vehicle only was subcutaneously (sc) administered to the animals daily for 20 days, and all rats were sacrificed 24 h after the last treatment. The adrenal glands were dissected, rapidly frozen, and stored at −80 °C for subsequent analyses.

### Catecholamine Quantification by Liquid Chromatography–Tandem Mass Spectrometry Analysis

For catecholamine (*i.e.*, norepinephrine and epinephrine) analysis, one adrenal gland was analyzed as reported by Su and colleagues^97^ with modifications. In brief, the tissue was homogenized using a Tissue Lyser II (Qiagen, Hilden, Germany) in ice-cold methanol after weighing precisely (1 g of tissue in 4 mL of methanol) supplemented with an internal standard, dopamine-1,1,2,2-*d*_4_ hydrochloride. Then, to remove particulate matter, the adrenal homogenates were centrifuged at 20,817*g* for 20 min at 4 °C. The supernatant was evaporated to dryness under a stream of nitrogen. The dry pellet was reconstituted with 300 μL of water, vortex-mixed for 10 s, and 300 μL of chloroform–isopropanol (100:30, v/v) was added. After vortex-mixing for 2 min, the mixture was centrifuged at 1187*g* for another 5 min. The upper aqueous layer was filtered with a 0.2 μm filter (SRC grade, regenerated cellulose membrane filter, CHMLAB Group), and then, for norepinephrine and epinephrine analysis, 5 μL of the sample volume was injected. To obtain the chromatographic separation of the analytes, a column for HPLC Luna Omega 5 μm PS C18 100 Å was used (Phenomenex, California, USA). The model of the mass spectrometer used is API 3500 (AB Sciex, USA), equipped with an electrospray source and triple quadrupole analyzer, interfaced with a pump for the HPLC model EXION SL (Sciex, USA).

### Western Blotting

Snap-frozen adrenal glands were homogenized in a Tissue Lyser II (Qiagen, Hilden, Germany) in a cold lysis buffer (phosphate-buffered saline, pH 7.4, added with 1% Nonidet P-40 and with a protease cocktail inhibitor), then sonicated, and centrifuged to remove particulate matter. After quantification, equal amounts of proteins for each sample were loaded into polyacrylamide electrophoresis gel and then electroblotted to a nitrocellulose membrane. For immunoblot detection, the filter was cut, and membranes were blocked on an orbital shaker at room temperature in PBS with added 0.1% Tween 20 and 10% nonfat dried milk. Successively, the 25 kDa part of the filter was incubated with mouse monoclonal antibody against PNMT, while in parallel, the 37 kDa part of the filter was incubated with a rabbit polyclonal antibody against GAPDH, as the housekeeping protein; both antibodies were incubated overnight at 4 °C. After extensive washing, the filters were incubated with an antimouse and antirabbit horseradish peroxidase-conjugated secondary antibody, respectively. After washing, bound antibodies were detected with the ECL method. A Chemi-Doc TM XRS+ system (Bio-Rad, Segrate, Italy) was used to acquire chemiluminescent signals, while Image Lab TM software, version 3.0 (Bio-Rad, Segrate, Italy), was used for their quantification. The mean control value within a single experiment was set to 100, and all other values were expressed as percentage. The values of controls from different experiments were all within 10%.

### Statistical Analyses

The quantitative data obtained by the catecholamine experiments were analyzed by inferential statistical analysis in accordance with the experimental protocols and the data (*i.e.*, Student’s *t*-test). *p* ≤ 0.05 was considered significant. Analyses were performed using GRAPHPAD PRISM, version 4.00 (GraphPad Inc., La Jolla, Calif., USA).

### PDB ID Codes

PNMT: 4MIK.

PNMT (cocrystallized with norepinephrine and S-adenosyl-l-homocysteine): 3HCD 5α-R2: 7BW1.

5βR: 3G1R.

## Supporting Information Available

Amino acid residues included in the RBS of finasteride and the corresponding PBS amino acid residues, steric clashes between amino acids of hPNMT and FIN, most relevant H-bonds and water-mediated bridges between FIN and hPNMT, MCOTA on five possible model organisms, RMSD vs the time profile for the 200 ns MD trajectory of P0–P3 and JIL, and representation of the electrostatic map generated for finasteride using the default options within the MOE software (PDF)
Residual PNMT activity (%) with 50 μM FIN (CSV)
Coordinates of the binding site residues (4.5 Å from the ligand) of the representative geometry obtained by clustering the MD trajectory of JIL (PDB)
P0 (PDB)
P1 (PDB)
P2 (PDB)
P3 (PDB)
Coordinates of the SPILLO-PBSS output for PNMT-FIT (PDB)

## Supplementary Material

jm0c02039_si_002.csv

jm0c02039_si_003.pdb

jm0c02039_si_004.pdb

jm0c02039_si_005.pdb

jm0c02039_si_006.pdb

jm0c02039_si_007.pdb

jm0c02039_si_008.pdb

## Abbreviations

5α-R: 5-alpha reductase
AGA: androgenetic alopecia
BPH: benign prostate hyperplasia
DHT: dihydrotestosterone
DMSO: dimethyl sulfoxide
FAERS: Food and Drug Administration Adverse Event Reporting System
MCOTA: multilevel cross-organism transferability analysis
MD: molecular dynamics
*NVT*: constant volume and temperature
*NPT*: constant pressure and temperature
OTPs: off-target proteins
PNMT: phenylethanolamine *N*-methyltransferase
PBSs: potential binding sites
RBS: reference binding site
RESP: restrained electrostatic potential
rhADA: recombinant human adenosine deaminase
rhAHCY: recombinant human adenosyl homocysteinase
RMSD: root-mean-squared deviation
SPILLO-PBSS: SPILLO potential binding sites searcher
