# Supplementary material for: Three-Dimensional Proteome-Wide Scale Screening for the 5-Alpha Reductase Inhibitor Finasteride: Identification of a Novel Off-Target
Source: J Med Chem. 2021 Apr 12;64(8):4553–66. doi: 10.1021/acs.jmedchem.0c02039 (PMC8154553; doi:10.1021/acs.jmedchem.0c02039)
Supplement: Supplementary file 1 — jm0c02039_si_001.pdf [file jm0c02039_si_001.pdf]

Supplementary Information

THREE-DIMENSIONAL PROTEOME-WIDE SCALE SCREENING FOR THE 5-ALPHA REDUCTASE INHIBITOR FINASTERIDE: IDENTIFICATION OF A NOVEL OFF-TARGET

Silvia Giatti [1#], Alessandro Di Domizio [1,2#], Silvia Diviccaro [1], Eva Falvo [1], Donatella Caruso [1], Alessandro Contini [3], Roberto Cosimo Melcangi [1*]

[1]Department of Pharmacological and Biomolecular Sciences, University of Milano, via Balzaretti 9, 20133 Milano, Italy

[2]SPILLOproject, via Stradivari 17, 20037, Paderno Dugnano, Milano, Italy (Website: www.spilloproject.com)

[3]Dipartimento Di Scienze Farmaceutiche, Università degli Studi di Milano, 20133 Milano, Italy

# S.G. and A.D.D. contributed equally to this work.

* Corresponding author: Roberto Cosimo Melcangi

Index

Supplementary Table 1.	S3
Supplementary Table 2.	S5
Supplementary Table 3.	S6
Supplementary Table 4.	S7
Supplementary Table 5.	S9
Supplementary Figure 1.	S11
Supplementary Figure 2.	S12
Supplementary Figure 3.	S13
Supplementary Figure 4.	S15

Supplementary Table 1. Amino acid residues included in the RBS of finasteride and the corresponding PBS amino acid residues in *Homo sapiens* PNMT (PDB code: 4MIK; UniProtKB AC: P11086), as identified by SPILLO-PBSS.

**Amino acid composition of the finasteride RBS**		**Amino acid composition of the finasteride PBS in *Homo sapiens* PNMT**
1st	LYS 1	LYS B 57
2nd	GLU 2	ASP B 267
3rd	ASP 3	ASP B 101
4th	TYR 4	TYR B 27
5th	TRP 5	PHE B 182
6th	MET 6	VAL B 187
7th	VAL 7	TYR B 35
8th	ILE 8	VAL B 269
9th	LEU 9	ASN B 39
10th	VAL 10	PRO B 82

11th	VAL 11	LEU B 103
12th	LEU 12	LEU B 229
13th	ILE 13	ALA B 186
14th	LEU 14	MET B 258
15th	ALA 15	PHE B 30
16th	PRO 16	PRO B 42
17th	MET 17	ASN B 38
18th	VAL 18	VAL B 266

Supplementary Table 2: Steric clashes between amino acids of hPNMT (PDB code: 4MIK) and finasteride are reported, along with the corresponding number of overlapping atoms, as found in the original SPILLO-PBSS output. In this analysis, a steric clash is generated when the distance between any two nuclei (one belonging to hPNMT and the other to finasteride) is smaller than 2.0 Angs.

**hPNMT amino acids overlapping with finasteride**	**Number of atoms overlapping with finasteride**
TYR B 35	8 atoms
ASN B 39	8 atoms
TYR B 40	7 atoms
ARG B 44	10 atoms
PHE B 182	4 atoms
TYR B 222	2 atoms

Supplementary Table 3. Most relevant H-bonds and water-mediated bridges[a] between finasteride (Fin) and hPNMT identified by the analysis of the last 20 ns of MD trajectory of P0

H-bond	Occ%
Lys57(NH_3)•••Fin(*N*-*t*Bu-C=O)	94.3
Fin(*N*-*t*Bu-NH)•••Tyr35(OH)	67.1
Fin(C^3=O)•••H-O-H•••Tyr27(C=O)	74.0
Fin(C^3=O)•••H-O-H•••Asn106(NH_2)	74.0

a. H-Bonds with occupancy higher than 30% are reported. Bridged H-bonds are mediated by a single water molecule bridging ligand and receptor.

Supplementary Table 4. Most relevant H-bonds and water-mediated bridges[a] between finasteride (Fin) and hPNMT identified by the analysis of the last 20 ns of MD trajectory of P1, P2 and P3.

Pose	H-bond	Occ%
P1	D101(CO_2)•••Fin(N^4H)	98.3
	Fin(C^3=O)•••H-O-H•••D101(CO_2)	87.2
	Fin(*N*-*t*Bu-C=O)•••H-O-H•••Y85(OH)	76.8
	Fin(C^3=O)•••H-O-H•••F102(NH)	40.2
P2	Fin(C^3=O)•••V159(NH)	96.6
	Fin(C^3=O)•••H-O-H•••D158(CO_2)	90.0
	P32(C=O)•••H-O-H•••Fin(*N*-*t*Bu-C=O)	80.9
	F182(C=O)•••H-O-H•••Fin(*N*-*t*Bu-NH)	72.5
P3	Fin(*N*-*t*Bu-C=O)•••Y27(OH)	99.7

	Y35(OH)•••Fin(N^4H)	87.5
	Fin(*N*-*t*Bu-C=O)•••Y40(OH)	42.8
	Fin(N^4H)•••H-O-H•••D101(CO_2)	31.5
	Fin(N^4H)•••H-O-H•••F102(NH)	31.5

a. H-Bonds with occupancy higher than 30% are reported. Bridged H-bonds are mediated by a single water molecule bridging ligand and receptor.

Supplementary Table 5. A multilevel cross-organism transferability analysis (MCOTA) on five possible model organisms identified *Rattus norvegicus* as the most suitable one to test *in vivo* the interaction between finasteride and PNMT.

**Model organism**	**I**<br><br>**BASIC CHECK**<br><br>Presence/absence of the PNMT gene/protein	**II**<br><br>**OVERALL PROTEIN SEQUENCE COMPARISON**<br><br>Protein sequence comparison with the '*Homo sapiens* PNMT' (P11086)		**III**<br><br>**LOCAL 3D-STRUCTURAL COMPARISON**<br><br>Analysis of the potential binding site (PBS) of the 'model organism PNMT'	**IV**<br><br>**PROTEOME-SCALE RANKING POSITION EVALUATION**<br><br>Ranking position of the 'model organism PNMT'
	**Search result**	**Identity (%)**	**Similarity (%)**	**SPILLO-PBSS score (%)**	**SPILLO-PBSS ranking position**
Caenorhabditis elegans	**ABSENT**	/	/	/	/
Danio rerio	**ABSENT**	/	/	/	/
Drosophila melanogaster	**ABSENT**	/	/	/	/
Mus musculus	**PRESENT**<br>UniProtKB: P40935	**80.7**	**86.1**	**83.493**<br>(vs. 83.498 obtained for *Homo sapiens* PNMT)	**7th out of 17927**<br>(vs. 6th obtained for *Homo sapiens* PNMT)<br>TARGET ZONE

Rattus norvegicus	**PRESENT** UniProtKB: P10937	**82.5**	**89.5**	**83.501** (vs. 83.498 obtained for *Homo sapiens* PNMT)	**5th out of 17927** (vs. 6th obtained for *Homo sapiens* PNMT) TARGET ZONE

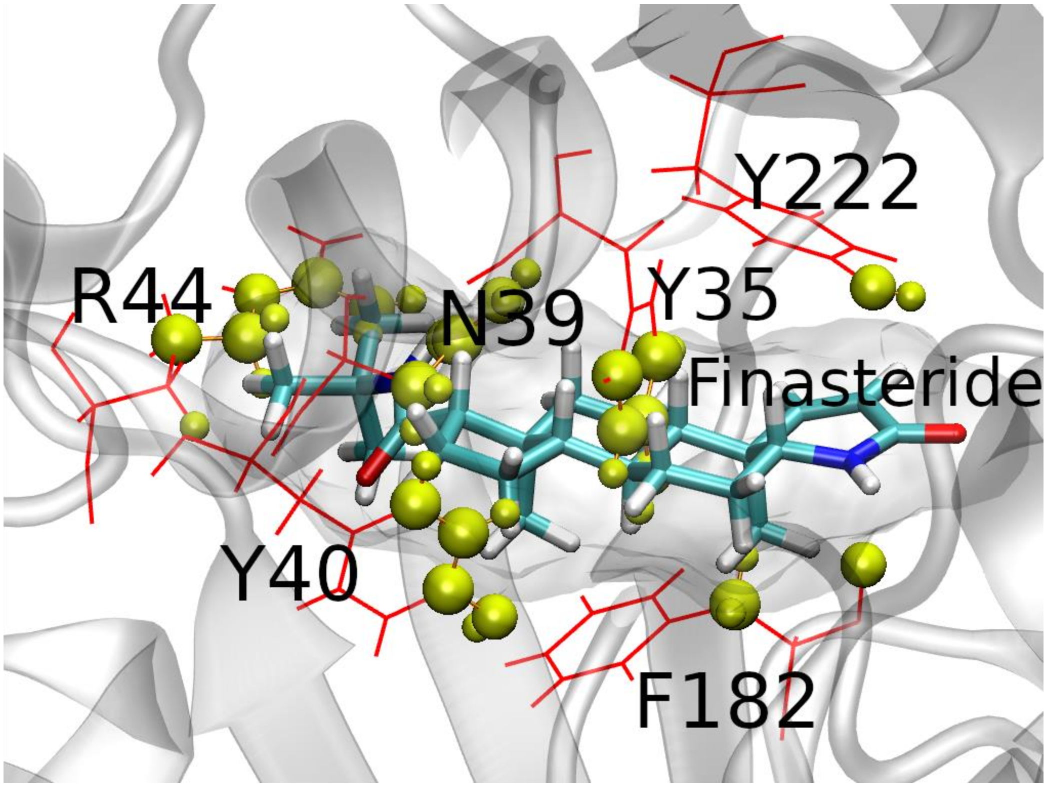

Supplementary Figure 1. Steric clashes between *Homo sapiens* PNMT (PDB code: 4MIK) and finasteride, as found in the original SPILLO-PBSS output. Amino acids listed in Supplementary Table S2 are reported in red, and the corresponding atoms giving rise to steric clashes are reported in yellow. A threshold distance of 2.0 Angs was here used as the distance below which the nuclei of any two atoms (belonging one to PNMT and the other to finasteride) give rise to a steric clash.

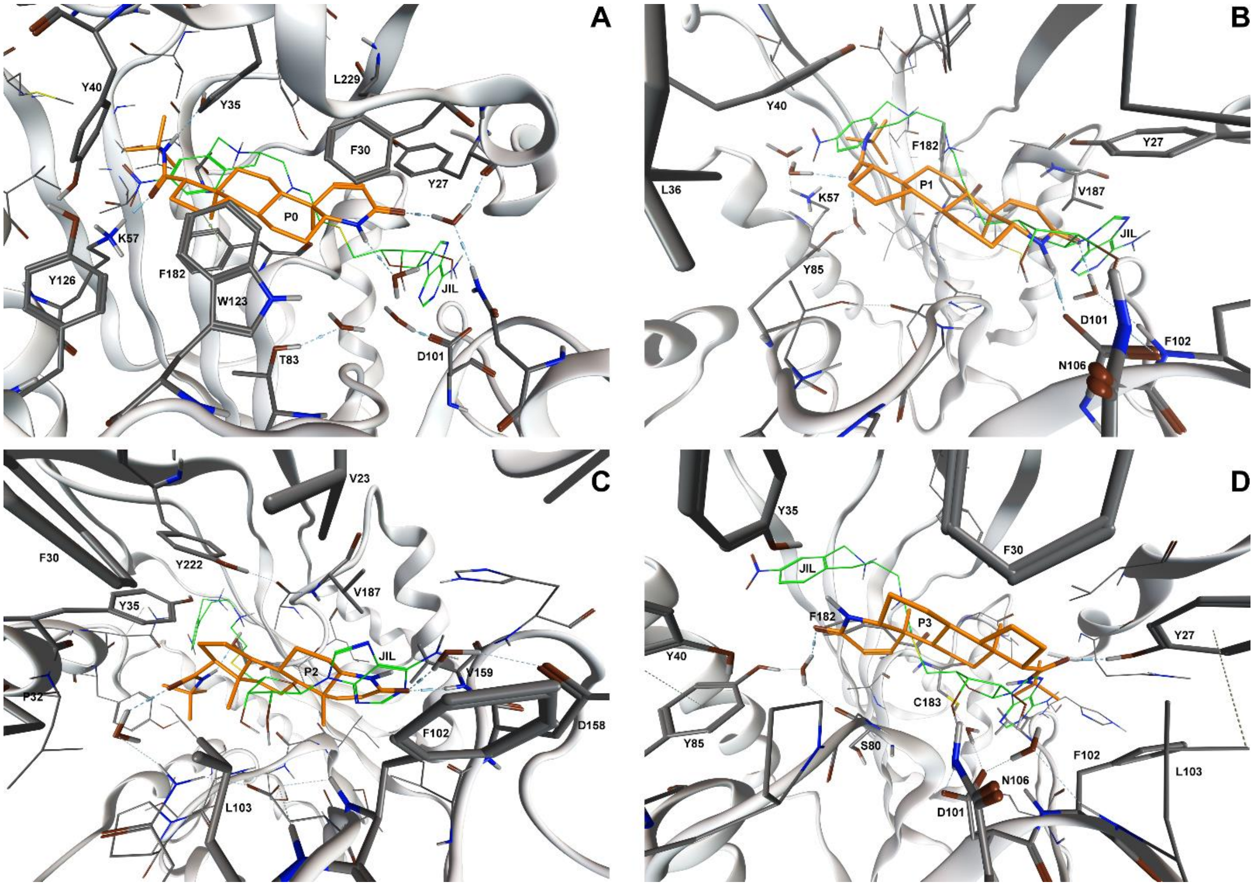

Supplementary Figure 2. Representative geometry obtained by cluster analysis of the final 20 ns of the MD trajectory of P0 (panel A), P1 (panel B), P2 (panel C) and P3 (panel D) poses. Human PNMT is represented by grey ribbons. Finasteride is depicted by tube representation with carbon atoms colored in orange. The reference crystallographic ligand JIL is depicted as green sticks.

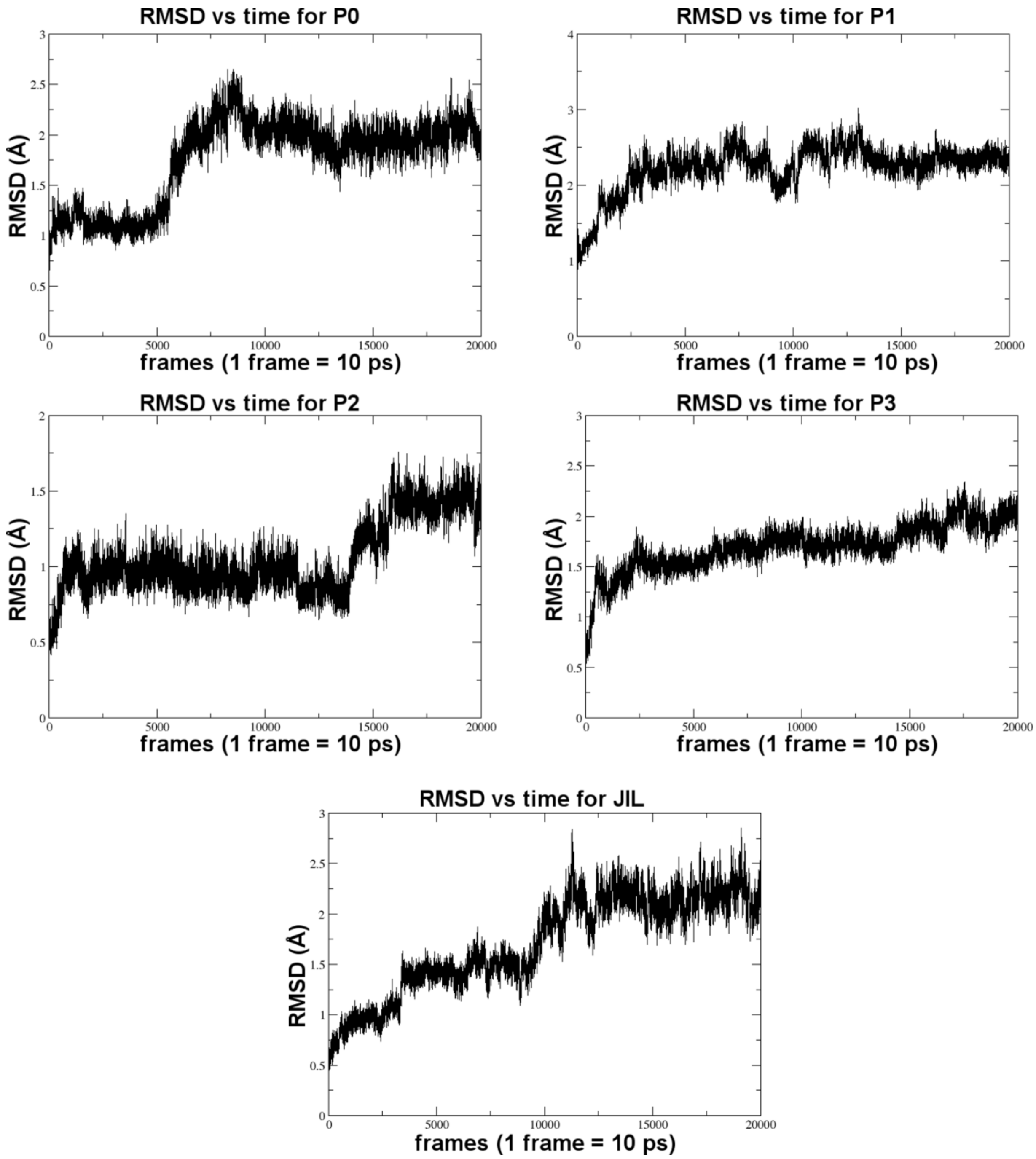

Supplementary Figure 3. RMSD vs time profile for the 200 ns MD trajectory of P0-P3 and JIL.

The backbone atoms of the binding site region (residues up to 5.0 Å from any of the

ligandatoms) were considered for the analysis.

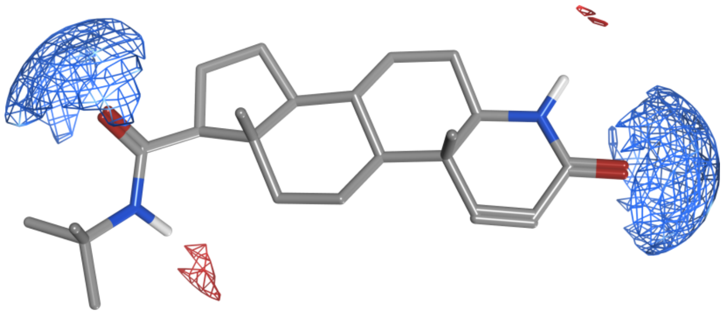

Supplementary Figure 4. Representation of the electrostatic map generated for finasteride using the default options within the MOE software. Blue and red grids represent negative and positive charge, respectively.
